# Evaluating the interplay between estrous cyclicity and flurothyl-induced seizure susceptibility in *Scn2a ^K1422E^* mice

**DOI:** 10.17912/micropub.biology.000850

**Published:** 2023-12-16

**Authors:** Dennis M. Echevarria-Cooper, Jennifer A. Kearney

**Affiliations:** 1 Northwestern University, Chicago, Illinois, United States

## Abstract

Recently, we demonstrated that *Scn2a ^K1422E^* female mice showed a distinct distribution of flurothyl-induced seizure thresholds. To evaluate whether the estrous cycle contributes to this effect, estrous cycle monitoring was performed in mice that had undergone ovariectomy, sham surgery, or no treatment prior to seizure induction. Ovariectomy did not affect the non-unimodal distribution of flurothyl seizure thresholds observed in *Scn2a ^K1422E^* females. Additionally, seizure thresholds were not associated with estrous cycle stage in mice that underwent sham surgery or in non-surgerized (intact) mice. Interestingly, intact *Scn2a ^K1422E^* females showed evidence of disrupted estrous cyclicity, an effect not previously described in a genetic epilepsy model.

**Figure 1. Evaluating estrous cyclicity and flurothyl-induced seizure susceptibility in Scn2a E/+ female mice following ovariectomy, sham surgery, or no surgery (intact):**
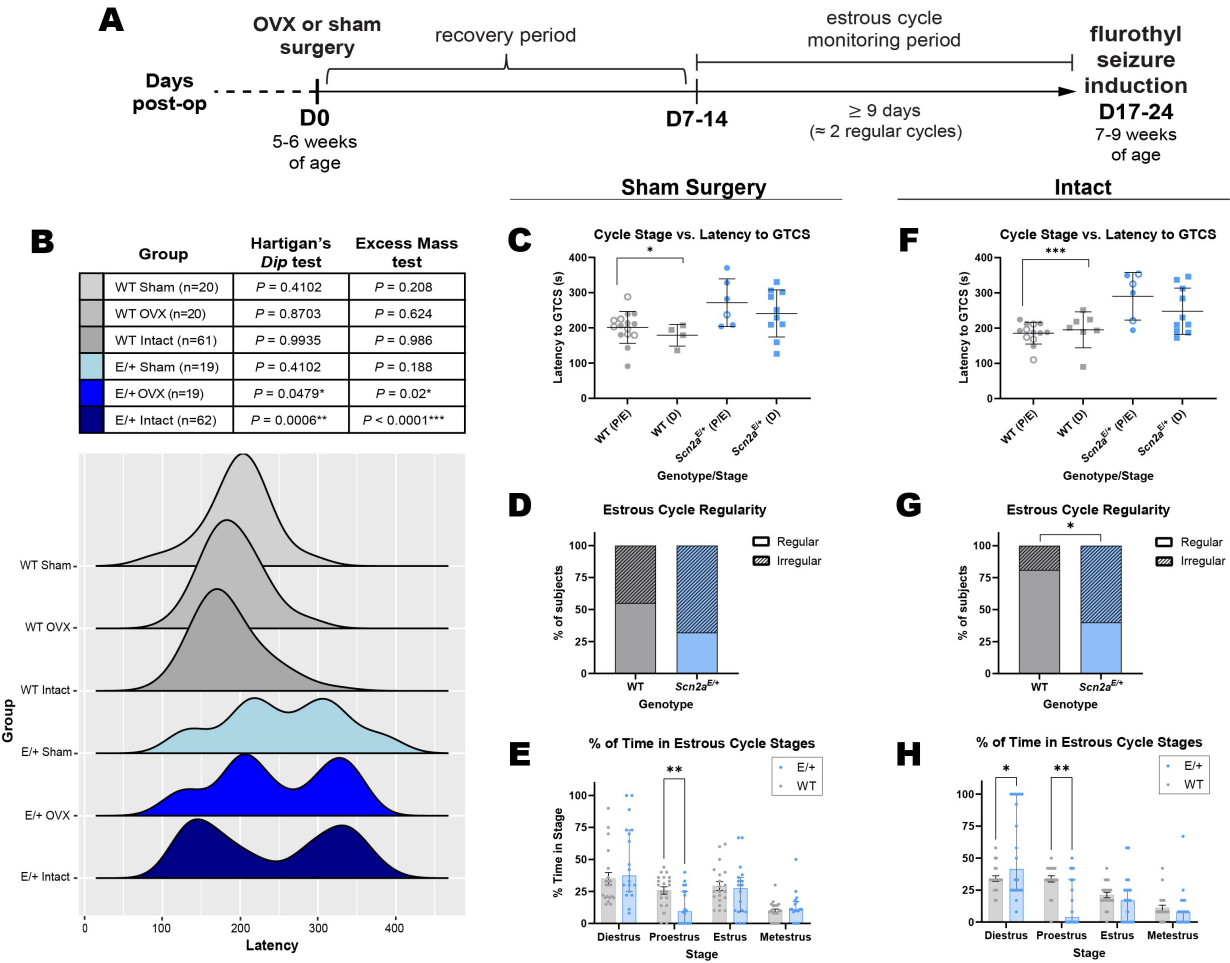
( **A** ) Schematic of experimental design for time points of surgery, estrous cycle monitoring and flurothyl seizure induction. Ovariectomy is abbreviated as OVX ( **B** ) Distributions of latencies to generalized tonic-clonic seizure (GTCS) were evaluated for unimodality/non-unimodality using hypothesis testing (top). Latency to first GTCS in WT and *Scn2a ^E/+^* female mice under different surgical conditions (bottom). Previously published data(Echevarria-Cooper et al., 2022) are replotted as WT Intact and E/+ Intact. Data from intact *Scn2a ^E/+^* females had a non-unimodal distribution while intact WT females had a unimodal distribution. WT females that underwent either OVX or sham surgery also had unimodal distributions. *Scn2a ^E/+^* females that underwent OVX had a non-unimodal distribution similar to intact controls. *Scn2a ^E/+^* females that underwent sham surgery had a distribution that did not reach statistical significance for non-unimodality but is still significantly different from WT controls ( *P=* 0.0218, Kolmogorov-Smirnov test). ( **C** ) Latency to first flurothyl-induced GTCS in sham ovariectomized WT and *Scn2a ^E/+^* female mice during pro/estrus (abbreviated P/E; open circles: proestrus; closed circles: estrus) or diestrus (abbreviated D). Two-way ANOVA showed only a significant main effect of genotype [ *F* (1,31)=9.791, *P* =0.0038]. Post hoc-analysis showed that *Scn2a ^E/+^* females in pro/estrus had an elevated threshold for GTCS compared to stage-matched WT controls (WT: 201.3 ± 45.2 s, *Scn2a ^E/+^* : 271.0 ± 67.0 s, ^*^ *P=* 0.0268; Sidak’s post-hoc test). Symbols represent individual mice, horizontal lines represent mean, and error bars represent SD; *n* = 4-15/stage/genotype. ( **D** ) Percent of sham ovariectomized WT and *Scn2a ^E/+^* female mice ( *n =* 19-20 per genotype) with regular vs irregular estrous cycles (WT: 45.0% irregular, *Scn2a ^E/+^* :68.4% irregular). Proportion of mice with irregular cyclicity was not significantly different between genotypes ( *P=* 0.2003, Fisher’s Exact test). Irregular cyclicity was defined as having a cycle length (average time to progress from one stage of estrus to the next) that exceeded 7 days or spending more than 50% of the monitoring period in any given stage. ( **E** ) Percent of monitoring time spent in each stage differed between sham *Scn2a ^E/+^* and WT mice [F(1.744, 62.77)=15.00, *P* <0.0001, two-way repeated measures ANOVA], with *Scn2a ^E/+^* mice spending less time in proestrus than WT mice ( ^*^ *P=* 0.0084; Sidak’s post-hoc test). ( **F** ) Latency to first flurothyl-induced GTCS in untreated WT and *Scn2a ^E/+^* female mice during pro/estrus (abbreviated P/E; open circles: proestrus; closed circles: estrus) or diestrus (abbreviated D). Two-way ANOVA showed only a significant main effect of genotype [ *F* (1,32)=18.47, *P* =0.0002]. Post hoc-analysis showed that *Scn2a ^E/+^* females in pro/estrus had an elevated threshold for GTCS compared to stage-matched WT controls (WT: 185.5 ± 30.6 s, *Scn2a ^E/+^* : 290.0 ± 67.0 s, ^***^ *P=* 0.0006; Sidak’s post-hoc test). Symbols represent individual mice, horizontal lines represent mean, and error bars represent SD; *n* = 6-13/stage/genotype. ( **G** ) Percent of sham untreated WT and *Scn2a ^E/+^* female mice ( *n =* 20-21 per genotype) with regular vs irregular estrous cycles (WT: 19.1% irregular, *Scn2a ^E/+^* :60.0% irregular). Proportion of *Scn2a ^E/+^* female mice with irregular cyclicity was significantly greater compared to WT ( ^*^ *P=* 0.0109, Fisher’s Exact test). Irregular cyclicity was defined as having a cycle length (average time to progress from one stage of estrus to the next) that exceeded 7 days or spending more than 50% of the monitoring period in any given stage. ( **H** ) Percent of monitoring time spent in each stage differed between intact *Scn2a ^E/+^* and WT mice [F(1.822,71.08)=20.40, *P* <0.0001, two-way repeated measures ANOVA], with *Scn2a ^E/+^* mice spending more time in diestrus ( ^*^ *P=* 0.0148; Sidak’s post-hoc test) and less time in proestrus ( ^**^ *P=* 0.0011; Sidak’s post-hoc test) compared to WT mice.

## Description

Genetic variants in *SCN2A* , encoding the Na _V_ 1.2 voltage-gated sodium channel, are associated with a range of neurodevelopmental disorders (NDD) including severe epilepsy syndromes and autism spectrum disorder (ASD) (Sanders et al., 2018) . Phenotypic variability observed across *SCN2A* -related NDD can be partially attributed to distinct changes in the biophysical properties of mutant channels (Ben-Shalom et al., 2017; Wolff et al., 2017; Berecki et al., 2022) . However, recurrent and inherited variants show heterogeneity even among individuals with the same variant, suggesting that phenotype expressivity may be subject to modifying factors (Syrbe et al., 2016) . The variant *SCN2A* -p.K1422E is associated with infant-onset developmental delay, infantile spasms, and features of ASD. We previously demonstrated that both male and female *Scn2a ^K1422E^* heterozygous mice (abbreviated as *Scn2a ^E/+^* going forward) had a higher threshold for flurothyl-induced generalized tonic-clonic seizure (GTCS) compared to WT (Echevarria-Cooper et al., 2022) . Within that initial data set, we also noted that the cumulative distribution of latencies to GTCS in *Scn2a ^E/+^* females was significantly different from the other groups (Echevarria-Cooper et al., 2022) . This effect was subsequently replicated in two additional cohorts of *Scn2a ^E/+^* and WT females (Echevarria-Cooper et al., 2022) . This is suggestive of a potential genotype-dependent interaction with a sex-specific modifying factor. Furthermore, sex differences have been reported in a number of NDD including epilepsy and ASD (Werling and Geschwind, 2013; Jacquemont et al., 2014; Savic, 2014) .

Epilepsy represents a diverse group of conditions for which differences between males and females can vary across different types of seizures (McHugh and Delanty, 2008; Savic, 2014; Christian et al., 2020a; Reddy et al., 2021) . The neurobiological basis for sex differences in seizure susceptibility is likewise varied and the subject of ongoing research (Christian et al., 2020a; Reddy et al., 2021) . It has been widely documented that women with epilepsy often show a cyclical pattern of altered seizure susceptibility during specific phases of the menstrual cycle (Newmark and Penry, 1980; Reddy, 2004; Herzog and Fowler, 2008) . This pattern of “catamenial epilepsy” is a neuroendocrine condition that affects between 25-75% of women with epilepsy (Reddy, 2009) . Additionally, menstrual disorders are more common in women with epilepsy than in the general population (Herzog, 2008) . The association between seizure susceptibility and the menstrual cycle can be attributed to fluctuations in hormones and corresponding changes in neurosteroid levels (Herzog, 1999; Reddy, 2004; Christian et al., 2020a; Reddy et al., 2021) . Rodent models have been used extensively to examine this association, revealing complex, sometimes contradictory effects of ovarian hormones and neurosteroids on seizure susceptibility (Finn and Gee, 1994; Woolley, 2000; van Luijtelaar et al., 2001; Scharfman et al., 2005; D’Amour et al., 2015; Li et al., 2020; Reddy et al., 2021). In general, estradiol is proconvulsant and progesterone (along with its neurosteroid derivative allopregnanolone) is anticonvulsant (Velíšková and DeSantis, 2013; Christian et al., 2020a; Reddy et al., 2021). Woolley and colleagues showed previously that neither estradiol nor progesterone affect susceptibility to flurothyl-induced seizures in ovariectomized rats (Woolley, 2000) . However, effects of the ovarian hormones on seizure susceptibility have not been evaluated in the context of an epilepsy-associated genetic variant, and it is possible that the K1422E variant may affect sensitivity to ovarian hormones. The nuclear estrogen receptor alpha is a transcription factor that is activated by endogenous estrogens (e.g. estradiol) and has been shown to target *Scn2a* in sexually dimorphic brain regions (Gegenhuber et al., 2022) . Based on these findings, we hypothesized that ovarian sex hormones affect susceptibility to flurothyl-induced seizures in *Scn2a ^E/+^* female mice.

To test our hypothesis, we sought to answer two essential questions. First, we wanted to know if circulating ovarian sex hormones are necessary to observe the non-unimodal distribution of flurothyl seizure thresholds in *Scn2a ^E/+^* mice. Second, we wanted to know if flurothyl seizure thresholds are associated with a particular stage of the estrous cycle. To evaluate whether the estrous cycle is associated with flurothyl seizure thresholds in *Scn2a ^E/+^* mice, we performed ovariectomy (OVX) or sham surgery. A schematic of the experimental design is shown in **Figure 1A** . After a recovery period of 7-14 days, we performed daily monitoring of the estrous cycle. Flurothyl was used to induce GTCS in WT and *Scn2a ^E/+^* mice 17-24 days after surgery (7-9 weeks of age). Specifically, we wanted to compare the distribution of latencies to GTCS in surgically treated mice against historical data from untreated mice (Echevarria-Cooper et al., 2022) . We reasoned that if circulating ovarian sex hormones were affecting flurothyl-seizure threshold, then removing these hormones via OVX would collapse the distribution observed in *Scn2a ^E/+^* females such that it would be more similar to the distributions observed in WT females or *Scn2a ^E/+^* males (Echevarria-Cooper et al., 2022) . In order to statistically evaluate distributions for unimodality/non-unimodality, we used Hartigan’s *dip* test and the multimode test proposed by Ameijeiras-Alonso and colleagues (Hartigan and Hartigan, 1985; Ameijeiras-Alonso et al., 2019) . The historical data from non-surgerized (intact) *Scn2a ^E/+^* females had a non-unimodal distribution, while historical data from intact WT females had a unimodal distribution ( **Figure 1B** ). Surgically treated (OVX and sham) WT females also had unimodal distributions of GTCS latencies similar to intact controls ( **Figure 1B** ). Contrary to our hypothesis, OVX did not affect the distribution of GTCS latencies in *Scn2a ^E/+^* females; the distribution representing this group was non-unimodal and significantly different from WT as determined by Kolmogorov-Smirnov test comparing cumulative distributions ( **Figure 1B ;** *P=0.0240* ). The above data suggest that ovarian sex hormones do not significantly contribute to flurothyl seizure thresholds in WT or *Scn2a ^E/+^* mice. *Scn2a ^E/+^* females that underwent sham surgery had a distribution that did not reach statistical significance for non-unimodality, although it was still significantly different from WT controls as determined by Kolmogorov-Smirnov test comparing cumulative distributions ( **Figure 1B** , *P=* 0.0218). For *Scn2a ^E/+^* mice undergoing surgery, modes of the distributions are less distinct likely due to the smaller group sizes and/or effects of surgery. However, this does not impact the statistical testing, which focused simply on unimodality versus non-unimodality.

Estrous cycle monitoring in surgically treated mice served a dual purpose. First, it allowed us to evaluate the success of OVX versus sham surgeries. Blind calls of surgical condition based on estrous cycle monitoring were nearly 100% accurate, with the exception of a single subject which was excluded. Second, it allowed us to evaluate whether flurothyl seizure threshold is associated with a particular stage of the estrous cycle in *Scn2a ^E/+^* mice that underwent sham surgery. We grouped subjects based on the estrous cycle stage determined on the day of flurothyl seizure induction. For the purposes of analysis, we compared stages that are estradiol-dominant (proestrus and estrus) with diestrus (progesterone-dominant) (Broestl et al., 2018; Li et al., 2020) . Two-way ANOVA comparing average latency to GTCS in sham surgery *Scn2a ^E/+^* females and WT controls showed a significant main effect of genotype ( **Figure 1C** ) in agreement with our previous findings on flurothyl seizure threshold in *Scn2a ^E/+^* mice (Echevarria-Cooper et al., 2022) . There was no significant effect of estrous cycle stage or genotype-by-stage interaction ( **Figure 1C** ). Although the genotype effect appears to be driven by the pro/estrus groups such that *Scn2a ^E/+^* females had a higher threshold for GTCS (271.0 ± 67.0 s) compared to WT (201.3 ± 45.2 s; **Figure 1C** ), the WT diestrus group was underrepresented (n = 4). Group numbers were unequal when comparing estrous cycle stage because we opted to maximize the number of subjects undergoing seizure induction on the same day under identical conditions. However, due to the lack of effect in the OVX treatment condition, there was little incentive to round out these estrous stage group numbers. Ultimately, these findings provide convergent evidence that the ovarian sex hormones do not significantly affect flurothyl seizure thresholds in *Scn2a ^E/+^* mice. Other neurobiological mechanisms apart from the acute influence of sex hormones have been proposed to address sex differences in seizure susceptibility (Christian et al., 2020) . In this case, any proposed mechanism would have to account for why the distribution of seizure thresholds is uniquely non-unimodal in *Scn2a ^E/+^* females. One possibility is that variation in one or more modifier genes confers sex-specific protection to females such that there is an increased threshold for penetrance of the K1422E variant. This is similar to a hypothesis that has been proposed to explain the “female protective effect” in ASD (Virkud et al., 2009; Jacquemont et al., 2014; Gockley et al., 2015; Werling, 2016) . Another possibility is that female mice are differentially exposed to androgens in utero based on their intrauterine position relative to male siblings (Lathe, 2004) . Androgen exposure in utero is known to mediate early brain development and could influence seizure susceptibility in differentially exposed females (Baron-Cohen, 2002; Christian et al., 2020b).

As part of our estrous cycle monitoring in surgically treated mice, we evaluated overall cycle regularity. A subject was defined as having an irregular cycle if cycle length exceeded 7 days or if the subject spent more than 50% of time in any given stage (Li et al., 2016, 2020) . We noted that a proportion of both WT and *Scn2a ^E/+^* sham ovariectomized mice had irregular cyclicity (WT: 45.0%, *Scn2a ^E/+^* : 68.4%), likely because of surgical intervention ( **Figure 1D** ). The percent of monitoring time in each stage detailed in **Figure 1E** showed that *Scn2a ^E/+^* sham mice spent less time on average in proestrus relative to WT sham mice. In order to exclude the confounding factor of surgical trauma, we wanted to evaluate whether flurothyl seizure threshold is associated with a particular stage of the estrous cycle in an additional cohort of untreated mice. Similar to what was observed in sham surgery mice ( **Figure 1C** ), two-way ANOVA comparing average latency to GTCS in non-surgerized (intact) *Scn2a ^E/+^* females and WT controls showed only a significant main effect of genotype, but no significant effect of estrous cycle stage or genotype-by-stage interaction ( **Figure 1F** ). Again, this effect appears to be driven by the pro/estrus groups such that *Scn2a ^E/+^* females had a higher threshold for GTCS (290.0 ± 67.0 s) compared to WT (185.5 ± 30.6 s; **Figure 1F** ). Interestingly, while the proportion of sham surgery mice with irregular cyclicity was not significantly different between genotypes ( **Figure 1D** ), the proportion of intact *Scn2a ^E/+^* females with irregular cyclicity was significantly greater than WT (WT: 19.1%, *Scn2a ^E/+^* : 60.0% irregular; *P=* 0.0109, Fisher’s exact test; **Figure 1G** ). The percent of monitoring time in each stage differed between intact *Scn2a ^E/+^* and WT mice, with *Scn2a ^E/+^* spending less time in proestrus and more time in diestrus relative to WT intact mice ( **Figure 1H** ). However, there was no correlation between percentage of time in each stage and GTCS seizure latency. This suggests that disrupted estrous cyclicity may be causally associated with the K1422E variant.

Disrupted estrous cyclicity has been observed in other rodent models of epilepsy, but this is the first demonstration of such an effect in a pathogenic variant model, particularly *SCN2A* -related NDD (Edwards et al., 1999; Fawley et al., 2012; Li et al., 2016, 2018) . This is important because epilepsy is generally associated with an increased risk for reproductive endocrine disorders, such as disrupted menstruation (Herzog, 1999, 2008; Christian et al., 2020) . However, the degree of comorbidity between these conditions and *SCN2A ^-^* related NDD specifically has not been formally studied. It has been shown that circadian signals from the suprachiasmatic nucleus (SCN) to gonadotropin-releasing hormone (GnRH) neurons in the hypothalamus are required for estrous cyclicity (Loh et al., 2014; Miller and Takahashi, 2014) . Sleep disturbances are frequently reported in individuals with *SCN2A-* related NDD, highlighting a connection between *SCN2A* and circadian rhythms (Crawford et al., 2021) . Region-specific deficiency of *Scn2a* has been used to model sleep disturbances in mice and is associated with disrupted firing of SCN neurons and changes in the expression of circadian entrainment pathway genes (Ma et al., 2022) . This suggests a possible mechanism by which changes in *Scn2a* function could lead to disrupted estrous cyclicity. Overall, we believe the current study reflects a technically sound experimental approach to probe the questions raised by our hypothesis, and our results support rejection of our original hypothesis that circulating ovarian sex hormones affect seizure susceptibility in *Scn2a ^E/+^* mice. Importantly, in conducting this study, we discovered disrupted estrous cyclicity in *Scn2a ^E/+^* mice, highlighting the value of investigating sex-specific effects and estrous cycle in genetic epilepsies and NDD, as well as in the broader field of neuroscience.

## Methods

***Mice* .** Female heterozygous *Scn2a ^E/+^* and WT mice for experiments were obtained from the line *Scn2a ^em1Kea^* (MGI:6390565; MMRRC:069700-UCD), which is maintained as an isogenic strain on C57BL/6J (#000664, Jackson Laboratory, Bay Harbor, ME). Mice were maintained in a specific pathogen free barrier facility with a 14 h light/10 h dark cycle and access to food and water ad libitum. All animal care and experimental procedures were approved by the Northwestern University Animal Care and Use Committees in accordance with the National Institutes of Health Guide for the Care and Use of Laboratory Animals. Principles outlined in the ARRIVE (Animal Research: Reporting of *in vivo* Experiments) guideline were considered when planning experiments (Sert et al., 2020).

***Surgeries.*** Bilateral ovariectomy (OVX) or sham surgery was performed on female WT and *Scn2a ^E/+^* mice at 5-6 weeks of age as previously described (Souza et al., 2019) . For both surgeries, subjects were deeply anesthetized with a cocktail of ketamine and xylazine (100 mg/kg and 10 mg/kg, respectively, IP), followed by administration of preoperative analgesia (20 mg/kg meloxicam, SC) and local anesthesia for the primary incision site (infiltration using 20 µL of 0.2% lidocaine). A primary midline skin incision and bilateral body wall incisions were performed to access the ovaries. For OVX, the ovarian fat pads were exteriorized, a crush injury was induced in the uterine horns below the ovaries, and the ovaries were subsequently removed. For sham surgeries, no crush injury was induced, and the intact ovarian fat pads were re-internalized following identification of the ovaries. At the conclusion of surgery, sustained release buprenorphine was administered (1 mg/kg, SC) for post-operative analgesia and atipamezole hydrochloride was administered (1 mg/kg, SC) to reverse the effects of anesthesia. All surgically treated subjects were housed individually during recovery and estrous cycle monitoring.

***Estrous cycle monitoring.*** Estrous cycle monitoring was performed using a vaginal lavage protocol as previously described (Pantier et al., 2019) . Cycle monitoring was conducted from 0700 to 0800 daily. Vaginal cytology was assessed using light microscopy (Olympus CX33 Biological Microscope) and representative images of each sample at 10x magnification were captured using an Apple iPhone 13 Pro (Camera app; 3x zoom) via the microscope eye piece. Estrous cycle stage was determined by evaluating the proportion of relevant cell types in a given sample as previously described (Nelson et al., 1982; Byers et al., 2012; McLean et al., 2012) . An additional classification, “unclear” was used to designate samples that contained significant cellular debris, making stage determination difficult. Cycle monitoring was performed in two cohorts of female WT and *Scn2a ^E/+^* mice beginning at 5-8 weeks of age. The first cohort consisted of mice that underwent either OVX or sham surgery ( *n* = 19–20 per genotype and treatment, blinded to treatment). Subjects were allowed 7-14 days to recover from surgery before daily estrous cycle monitoring was performed leading up to, and on the day of, flurothyl seizure induction ( **Figure 1A** ). The monitoring period was at least 9 days for each for each subject, corresponding to approximately 2 cycles of average length (Nelson et al., 1982) . Estrous cycle monitoring data was used to make blinded calls of surgical condition based on estrous cyclicity (i.e., cycle regularity). A subject was defined as having an irregular cycle if cycle length (average time to progress from one stage of estrus to the next) exceeded 7 days or if the subject spent more than 50% of time in any given stage (Li et al., 2016, 2020) . A subject was blind called as having undergone OVX if it had an irregular cycle with a majority of samples determined to be “unclear” (Ng et al., 2010) . A single subject was excluded from analyses due to being blind called as receiving a sham surgery despite actually receiving OVX. The second cohort consisted of consisted of non-surgerized mice ( *n* = 20–21 per genotype) evaluated separately from the previous cohort. Daily estrous cycle monitoring was performed leading up to, and on the day of flurothyl seizure induction. The monitoring period was 12 days for each subject, corresponding to approximately 3 cycles of average length (Nelson et al., 1982) . Estrous cycle monitoring data was used to evaluate estrous cyclicity using the criteria defined above. Detailed estrous cycle monitoring data is available at https://doi.org/10.18131/pvcx7-p9x63 .

***Flurothyl seizure induction.*** Susceptibility to seizures induced by the chemoconvulsant flurothyl (Bis(2,2,2-trifluoroethyl) ether, Sigma-Aldrich, St. Louis, MO, USA) was assessed in two cohorts of female WT and *Scn2a ^E/+^* mice at 7-10 weeks of age. Flurothyl was introduced into a clear, plexiglass chamber (2.2 L) by a syringe pump at a rate of 20 µL/min and allowed to volatilize. Latency to first GTCS with loss of posture was recorded. The first cohort consisted of mice that underwent either OVX or sham surgery ( *n* = 19–20 per genotype and treatment). The second cohort consisted of non-surgerized mice ( *n* = 20–21 per genotype) evaluated separately from the previous cohort.

**Statistical analysis**

Distributions of latencies to flurothyl-induced GTCS were evaluated for unimodality/non-unimodality in six groups based on genotype and surgical condition using the R packages ‘diptest’ and ‘multimode’ (RStudio 4.2.0) (Ameijeiras-Alonso et al., 2021; Maechler, 2021; R Core Team, 2021) . Both analytical methods involve hypothesis testing in which unimodality represents the null hypothesis and a *P* -value of ≤ 0.05 resulted in rejection of the null hypothesis. *P* -values for all groups are shown in **Figure 1B** . Two distributions representing previously published data (Echevarria-Cooper et al., 2022) from untreated female WT and *Scn2a ^E/+^* mice are replotted as “WT Intact” ( *n* = 61) and “E/+ Intact” ( *n* = 62) in **Figure 1B** . These distributions were not previously evaluated specifically for unimodality/non-unimodality. Mice from these historical groups did not undergo estrous cycle monitoring. The remaining four distributions represent WT and *Scn2a ^E/+^* mice that underwent either OVX or sham surgery and subsequent estrous cycle monitoring. All other comparisons were performed using GraphPad Prism (v 9.4.1). Two-way ANOVA was used to look at the effect of genotype and estrous cycle stage on latency to GTCS in the sham surgery cohort and in a separate cohort of non-surgerized mice ( **Figure 1C &E).** Sidak’s post-hoc test was used to compare group means. Fisher’s exact test was used to compare irregular estrous cycle risk between genotypes in the sham surgery cohort and in a separate cohort of non-surgerized mice ( **Figure 1D &F).**
